# Pharmacoepigenomic Impact of Antihypertensive Drugs on miRNome and Proteome and Its Potential Influence on Health and Side Effects

**DOI:** 10.3390/cells14171359

**Published:** 2025-08-31

**Authors:** Samyukta Bhass, Moinak Banerjee

**Affiliations:** 1 Human Molecular Genetics Lab, Neurobiology and Genetics Division, BRIC-Rajiv Gandhi Centre for Biotechnology, Thiruvananthapuram 695014, India; 2 BRIC-Regional Centre for Biotechnology, NCR Biotech Science Cluster, Faridabad 121001, India

**Keywords:** antihypertensive drugs, pharmacoepigenetics, microRNA, adverse effects, hypertension, phenotype

## Abstract

Antihypertensive drugs are widely used for the treatment of hypertension, and the choice of drug and dosage is based on trial and error. The variability in drug response and adverse reactions leads to the poor adherence to treatment. Epigenetic modulation is one of the major mechanisms that may contribute to the variability in drug responses, and microRNAs (miRNAs) can serve as crucial epigenetic regulators and have also been reported to be associated with hypertension pathogenesis. The objective of this study is to investigate the regulatory effects of commonly used antihypertensive drugs on the endothelial miRNome in human aortic endothelial cells. We aim to integrate miRNA expression data with proteomic analyses to elucidate drug-induced molecular mechanisms relevant to hypertension treatment. Whole genome small RNA sequencing was performed, followed by whole proteome analysis using LC-MS/MS comparing between control and treated samples. The treatments induced significant differential regulation of several miRNAs and proteins; among these, a few reflected reverse relationships with miRNA regulation and protein expression. Certain miRNAs and their corresponding target proteins seem to distinguish between good therapeutic outcomes and potential side effects. This study unravels the potential role of drug-induced miRNAs in inducing post-transcriptional modifications to cause the differential expression of certain proteins that may induce not only therapeutic effects or drug side effects but can also indicate the potential for drug-repurposing in other diseases.

## 1. Introduction

Antihypertensive drugs are the mainstay therapy for hypertension, an eminent modifiable risk factor for numerous cardiovascular and cerebrovascular complications [1,2]. Patients receiving antihypertensive drugs are often prescribed in a “trial and error” manner by adjusting drug regimens iteratively to achieve optimal blood pressure control and also display significant variability in drug response [3,4].

Despite the availability of effective therapies, only about 40% of hypertensive patients achieve optimal blood pressure control, reflecting persistent barriers, such as complex treatment regimens and insufficient patient and healthcare system engagement [5]. Though proper treatment management of hypertension and blood pressure reduces the complications associated with conditions, like stroke, heart failure, and coronary diseases [6], studies have shown that only 21% of hypertensive individuals receiving the drug have positive therapeutic effect [7]. The drugs are also reported to cause adverse reactions, which leads to poor treatment adherence and treatment discontinuation [8]. Several studies across different health centers and hospitals have shown that antihypertensives can cause serious side effects [9]. Increased teratogenic effects are also reported to be associated with the administration of antihypertensive drugs [10,11]. These challenges underscore the limitations of generalized pharmacotherapeutic approaches and necessitate a deeper molecular understanding of how these drugs influence mechanisms including gene regulation.

In this regard, a class of small non-coding RNAs known as microRNAs (miRNAs), which regulate gene expression post-transcriptionally, have emerged as critical regulators of some main physiological pathways implicated in hypertension, including vascular development, RAAS pathway, NO production, and tubular transport [12]. Numerous studies have shown the differential regulation of miRNA associated with hypertension condition, highlighting their potential as modulators of disease pathophysiology or biomarkers [13]. While dysregulation of miRNAs has been well-studied in the pathogenesis of hypertension, the influence of antihypertensive drugs on miRNA regulation and contribution of miRNA regulation due to antihypertensive drugs in hypertension therapy or development of drug adverse reactions remains largely unexplored. To our knowledge, this is the first study to investigate the influence of antihypertensive drugs on miRNA regulation and their downstream proteomic impact. While miRNA dysregulation has been widely studied in hypertension pathogenesis, the effects of commonly prescribed antihypertensive medications on miRNA expression and the resulting proteomic changes remain largely unexplored. This gap limits our understanding of the molecular mechanisms underlying therapeutic responses and adverse drug reactions in hypertension management. Addressing these gaps is critical for developing personalized treatment strategies and improving patient outcomes. Understanding the molecular interplay among antihypertensive drugs, miRNA expression, and their downstream targets is crucial in developing personalized approaches to hypertension management and other complications associated with elevated blood pressure as well as in minimizing side effects-induced by the drugs. The study was designed to assess changes in the endothelial miRNome upon treatment with antihypertensive drugs and to correlate these changes with downstream protein expression alterations, using small RNA sequencing and LC-MS/MS proteomics in human aortic endothelial cells.

The study aims to investigate the role of antihypertensive drugs in miRNA modulation thus contributing to the treatment responses. The gene-silencing and post transcriptional events are marked by the level of miRNA and their target mRNA. Thus, the study assessed the level of miRNA post antihypertensive treatment. The validation of target gene expression at the protein level was determined, as the differential expression of certain proteins can have a biological significance in therapeutic response as well as in adverse effects due to the drug.

## 2. Materials and Methods

### 2.1. Study Design

The study aims to investigate how antihypertensive drugs modulate miRNA expression and their target proteins, affecting therapeutic responses. The analysis of differentially expressed miRNAs and protein expression following drug treatment was performed using an in vitro cell culture system. Validated miRNA targets were identified, and protein changes were assessed to understand the biological impact related to efficacy and side effects. The entire methodology is shown in a flowchart (Figure S1).

### 2.2. Cell Culture

The human aortic endothelial cells (HAECs; LONZA, CC2535), were grown using the Endothelial cell growth medium 2 (LONZA, Basel, CH, EGM™-2 Endothelial Cell Growth Medium-2 Bullet Kit™, CC-3162) at 37 °C with 5% CO_2_ in a humidified incubator. The seeding density in a 6-well plate was 3 × 10^5^ cells per well, with each plate dedicated to a specific treatment concentration and timing. All experimental protocols were carried out in triplicate.

### 2.3. Antihypertensive Drug Treatment

One among the most used drugs from each class of antihypertensives was selected for the treatment in the study. This includes Amlodipine (a dihydropyridine calcium channel blocker), Enalapril (an ACE inhibitor), Telmisartan (an angiotensin II type 1 receptor blocker), and Metoprolol (a cardio-selective beta blocker) (Sigma- Aldrich, St. Louis, MO, USA). The drugs were dissolved in dimethyl sulfoxide (DMSO), and thus, DMSO was the control experiment. The final concentration of DMSO at 0.20% in EGM-2 medium was maintained. All the drugs were treated at 5 μm, 10 μm, 20 μm, 50 μm, and 100 μm concentrations, and the appropriate concentrations of antihypertensive drugs were selected based on the MTT (3-(4,5-dimethylthiazol-2-yl)-2,5-diphenyltetrazolium bromide) cell viability assay. Treatment was performed for 6 h and 24 h (except for MS, which has a half-life of less than 10 h) [3], and the concentrations, which were equivalent to therapeutic concentrations with more than 80% of the cells being viable, were selected for further study (Figure S2). A comprehensive time-course analysis for all concentrations and time points was not performed for the current study. Selection of the treatment concentration and duration for the current study was guided by initial pilot studies, including expression studies [14], rather than systematic survey of all possible conditions. These preliminary data indicated negligible effects for telmisartan, justifying the omission of the 6-h time point from later analysis.

### 2.4. RNA Extraction and Small RNA Sequencing

RNA was extracted from the cell lines following treatment with selected concentration of the drugs using the total RNA extraction reagent, RNA isoplus, (Takara Bio, Kusatsu, Japan) as per the manufacturer’s instructions. The RNA with an RNA integrity (RIN) number ≥ 8 was included for the assay. Small RNA sequencing was performed using the Illumina NovaSeq X plus platform for selected drug treatment concentrations. Small RNA libraries were prepared following the standard protocol. Raw fastq files were generated following sequencing, de-multiplexed, and subjected to quality control using the FastQC software (https://www.bioinformatics.babraham.ac.uk/projects/fastqc/). The low-quality bases at the 3′ end was trimmed, retaining the reads between 17 and 30 nucleotides. Reads were collapsed and aligned to the reference miRbase database using default alignment parameters. Identification and quantification of the known miRNAs was followed by differential expression analysis. miRNAs with a *p* value < 0.05 were considered for the study.

The target genes of the significant differentially regulated miRNAs were derived from the miRwalk database [15] using the filters that retained only validated genes. The miRNA binding site was the 3′UTR with a binding probability score (bindingp) of ≥0.95, representing the probability that the miRNA–target interaction is functionally relevant. 

### 2.5. Protein Isolation and Whole Proteome Analysis

A total of 3 × 10^5^ cells were seeded in six-well plates for treatment studies. Protein isolation and whole proteome analysis were conducted following the protocol described earlier [16]. It is further described briefly. Upon exposure to experimental conditions, the cells were washed once with ice-cold phosphate-buffered saline (PBS), scraped into fresh cold PBS, and centrifuged at 2000 rpm for 5 min to pellet the cells. The pellet was then suspended in 50 µL of RapiGest™ Protein Extraction Buffer containing 0.5% of RapiGest™ in 50mM ammonium bicarbonate buffer and subjected to sonication to fragment nucleic acids. To enhance cell lysis, two cycles of rapid liquid nitrogen freezing and thawing at 4 °C were applied to the samples for three minutes each cycle. Debris was removed with centrifugation for 10 min at 12,000 rpm at 4 °C, followed by the transfer of the supernatants containing proteins to new tubes, which were stored at −80 °C for subsequent use. The Bradford method, employing bovine serum albumin standards, was used to estimate the protein content. Desalting was carried out using Amicon Ultra centrifugal filters (0.5 mL capacity, 3000 MWCO, Millipore, Burlington, MA, USA UFC500324) in accordance with manufacturer protocols.

Protein lysates were enzymatically digested with trypsin, and analysis of peptides were carried out on an UltiMate™ 3000 RSCLC nano Ultra High-Performance Liquid Chromatography (UHPLC) system (Thermo Fisher Scientific, Waltham, MA, USA) combined with an Orbitrap Eclipse Tribrid Mass Spectrometer (Thermo Fisher Scientific, Waltham, MA, USA). An EASY-Spray PepMap C18 Column (100 Å, 2 µm, 75 µm x 500 mm; Part No.ES903, Thermo Fisher Scientific, Waltham, MA, USA) was utilized for separation and held at a temperature of 40 °C. Loading of 300ng peptide obtained from each individual sample was performed for trapping and separation at a 250 nL/min flow rate with the help of a binary gradient that was made up of solvent A (0.1% formic acid in water) and solvent B (0.1% formic acid in acetonitrile). This was applied according to programmed steps: initial equilibration with 5% B for 5 min, ramping up to 45% B over the next 90 min, then further increases to 60% B (110 min), 95% B (120 min), holding for 10 more minutes which then returned to B at 5% in 131 min. Positive ion mode and nano-spray ionization (capillary voltage 1400 V) were used for detection. Peptide spectrum matches were validated with a stringent false discovery rate (FDR ≤ 1%), and protein identification required a minimum of one unique peptide. Carbamidomethylation was set as static modification, whereas dynamic modifications comprised of oxidation at the methionine and phosphorylation at the serine, threonine, and tyrosine. Proteome Discoverer v3.0.1.27 (Thermo Fisher Scientific, Waltham, MA, USA) was used for processing and analysis of the data.

Processing and analysis of the Mass Spectrometry Elevated Collision Energy (MSE) spectra were carried out with Proteome Discoverer version 3.0.1.27 to facilitate protein identification and quantification. The processing of data included lock mass correction post-acquisition to enhance mass accuracy, applying noise filtering thresholds of 150 and 30 counts for low and high energy ions, respectively, during scanning to improve signal quality. Database interrogations against the UniProt human proteome were employed for peptide identification, applying a stringent FDR cutoff of ≤1.0% to ensure high-confidence results. The standards for protein identification included a minimum of one fragment ion match per peptide, three fragment ion matches per protein, and at least one peptide match. The datasets were evaluated using the Hi-N algorithm to resolve peptide assignment conflicts by taking the average intensity measurements of the three most abundant unique peptides associated with each protein. Peptide abundance was quantified based on the cumulative intensity of all constituent peptide ions, whereas protein abundance was assessed by calculating the average intensity of the most abundant peptides, providing a representative value for every individual protein. The rankings of peptide abundance were assigned by integrating the values from all analytical runs, supported by precise alignment and the elimination of missing data. This detailed procedure supports reliable peptide selection and robust relative protein quantification across repeated analyses.

LC-MS/MS was carried out on three separate biological replicates, each comprising two technical replicates per biological replicate. The ratios of protein abundance were calculated as treated relative to control (treated/control), where a ratio greater than one indicated upregulation and less than one indicated downregulation in the treated samples compared to controls. Proteome Discoverer version 3.0.1.27 was used consistently for proteomics data processing and analysis.

### 2.6. Integrated Analysis of the Coexpression Network of miRNA Target Genes and Target Proteins from Proteome Data

The datasets were compared, and the overlaps were identified using SR plot, a web-based tool [17]. The expression of the validated target genes at the protein level was analyzed, and the miRNA-regulated proteins were investigated for their potential role in treatment based on genes associated with hypertension and related conditions from the GWAS catalog and literature search. The potential role of the proteins in the reported adverse drug reactions was investigated based on the genes associated with the side effects according to the Human Phenotype Ontology database (HPO) [18] and literature search. Network analysis of drug-induced differentially expressed miRNAs and their respective target genes was conducted using the online platform miRNet [19]. The input given was the differentially expressed miRNAs after each treatment. The resulting network diagram with target genes was color coded and highlighted for the validated target genes with 0.95bindingp and 3′UTR binding site, based on their possible association with hypertension (nodes highlighted in red), drug side effects (nodes highlighted in cyan), and those that were reflected at the protein level (nodes highlighted in red).

## 3. Results

### 3.1. Differentially Expressed microRNAs

**Figure 1:**
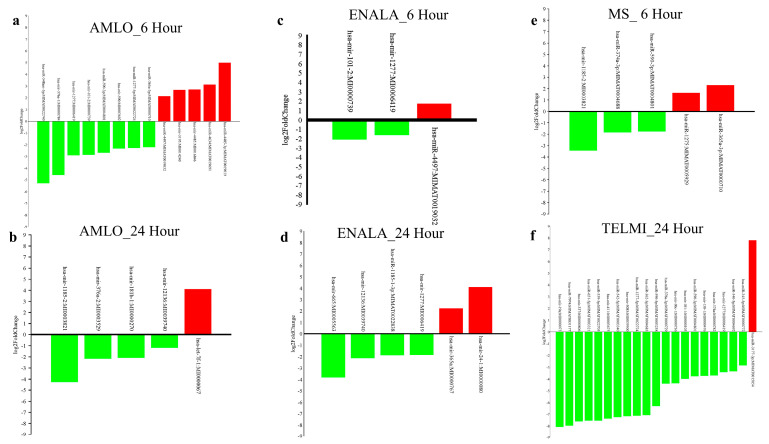
Up and down bar plot representing significantly upregulated and downregulated miRNAs after each treatment. (**a**) Following 6−h Amlodipine (AMLO) treatment, (**b**) following 24−h AMLO treatment, (**c**) following 6−h Enalapril (ENALA) treatment, (**d**) following 24−h ENALA treatment, (**e**) 6−h Metoprolol (MS) treatment, (**f**) 24−h Telmisartan (TELMI) treatment. (green bars represent downregulated miRNAs, red bars represent upregulated miRNAs).

The antihypertensive treatment led to the differential regulation of the number of miRNAs compared to the control (Figure 1). The in vitro treatment included drug concentrations of 5 μm Amlodipine (AMLO) and 5 μm Enalapril (ENALA), with a duration of 6 h and 24 h, 10 μm Metoprolol (MS) for 6 h, and 20 μm Telmisartan (TELMI) for 24 h. We observed that following 6 h of treatment with AMLO, 1546 miRNAs were differentially regulated, of which miRNAs hsa-miR-365a-3p:MIMAT0000710 (*p*-value: 0.028), hsa-miR-590-3p:MIMAT0004801 (*p*-value: 0.015), hsa-mir-376a-1:MI0000784 (*p*-value: 0.008), hsa-mir-590:MI0003602 (*p*-value: 0.021), hsa-mir-101-2:MI0000739 (*p*-value: 0.008), hsa-mir-1277:MI0006419 (*p*-value: 0.008), hsa-miR-548am-5p:MIMAT0022740 (*p*-value: 0.031), and hsa-miR-1277-5p:MIMAT0022724 (*p*-value: 0.040) showed significant downregulation and hsa-miR-4485-3p:MIMAT0019019 (*p*-value: 0.047), hsa-mir-4485:MI0016846 (*p*-value: 0.021), hsa-miR-4634:MIMAT0019691 (*p*-value: 0.012), hsa-mir-3195:MI0014240 (*p*-value: 0.031), and hsa-miR-4497:MIMAT0019032 (*p*-value: 0.032) showed significant upregulation (Figure 1a), and following 24 h of treatment, 1429 miRNAs were differentially regulated, of which hsa-mir-12136:MI0039740 (*p*-value: 0.033), hsa-mir-1185-2:MI0003821 (*p*-value: 0.045), hsa-mir-376a-2:MI0003529 (*p*-value: 0.047), and hsa-mir-181b-1:MI0000270 (*p*-value: 0.014) were significantly downregulated and hsa-let-7f-1:MI0000067 (*p*-value: 0.015) was significantly upregulated (Figure 1b). The 6-h ENALA treatment identified 1603 differentially regulated miRNAs, among which hsa-mir-101-2:MI0000739 (*p*-value: 0.014) hsa-mir-1277:MI0006419 (*p*-value: 0.035) were downregulated and hsa-miR-4497:MIMAT0019032 (*p*-value: 0.039) was upregulated significantly (Figure 1c), and 24-h treatment resulted in 1575 differentially regulated miRNAs, among which hsa-miR-1185-1-3p: MIMAT0022838 (*p*-value: 0.043), hsa-mir-12136:MI0039740 (*p*-value: 0.017), hsa-mir-1277:MI0006419 (*p*-value: 0.042), and hsa-mir-665:MI0005563 (*p*-value: 0.022) were significantly downregulated and hsa-mir-365a:MI0000767 (*p*-value: 0.023) and hsa-mir-24-1:MI0000080 (*p*-value: 0.025) were upregulated significantly (Figure 1d). MS treatment led to differential regulation of 1630 miRNAs, among which hsa-miR-590-3p: MIMAT0004801 (*p*-value: 0.049), hsa-mir-1185-2:MI0003821 (*p*-value: 0.035), and hsa-miR-374a-3p: MIMAT0004688 (*p*-value: 0.039) were significantly downregulated and hsa-miR-1275: MIMAT0005929 (*p*-value: 0.050) and hsa-miR-365a-3p: MIMAT0000710 (*p*-value: 0.010) were upregulated (Figure 1e). Following 24-h treatment with TELMI, 1393 miRNAs were differentially regulated, among which hsa-mir-3085:MI0039500 (*p*-value: 0.044), hsa-miR-590-3p:MIMAT0004801 (*p*-value: 0.026), hsa-mir-30c-1:MI0000736 (*p*-value: 0.029), hsa-mir-374a:MI0000782 (*p*-value: 0.024), hsa-mir-411:MI0003675 (*p*-value: 0.034), hsa-miR-340-5p:MI MAT0004692 (*p*-value: 0.025), hsa-miR-345-5p:MIMAT0000772 (*p*-value: 0.047), hsa-mir-101-1:MI0000103, hsa-mir-19a:MI0000073, hsa-miR-362-3p:MIMAT0004683, hsa-mir-138-1:MI0000476 (*p*-value: 0.042), hsa-mir-1277:MI0006419 (*p*-value: 0.029), hsa-mir-337:MI0000806 (*p*-value: 0.025), hsa-miR-590-5p:MIMAT0003258 (*p*-value: 0.015), hsa-miR-7974: MIMAT0031177 (*p*-value: 0.015), hsa-miR-651-5p:MIMAT0003321 (*p*-value: 0.027), hsa-miR-376a-3p:MIMAT0000729 (*p*-value: 0.010), hsa-miR-542-5p:MIMAT0003340 (*p*-value: 0.039), hsa-miR-1277-5p:MIMAT0022724 (*p*-value: 0.005), and hsa-miR-539-3p:MIMAT0022705 (*p*-value: 0.027) were significantly downregulated and hsa-miR-3177-3p:MIMAT0015054 (*p*-value: 0.024) was significantly upregulated (Figure 1f). The list of significantly regulated miRNAs (*p*-values < 0.05) with the respective fold change are given in Table S1.

**Figure 2:**
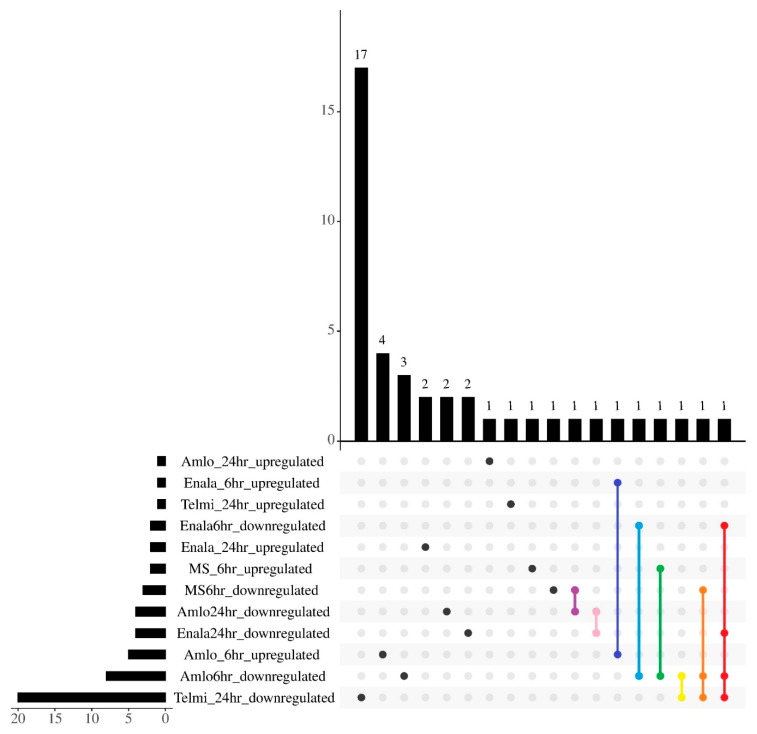
Upset plot visualizing the overlap and distinctness of differentially regulated miRNAs across various drugs and durations. The horizontal bar representing the number of differentially regulated miRNAs after each treatment; vertical bars represent the unique and overlapping miRNAs within the treatments. Here the black dots represent the unique miRNAs. The purple line represents the common downregulated miRNAs between 6-h Amlodipine (AMLO) and Metoprolol (MS) treatments; the pink line represents common downregulated miRNAs between 24-h AMLO and Enalapril (ENALA) treatments; the indigo line represents common upregulated miRNAs between 6-h AMLO and ENALA treatments; the blue line represents common downregulated miRNAs between 6-h AMLO and ENALA treatments; the green line represents overlap between 6-h AMLO downregulated and 6-h MS upregulated miRNAs; the yellow line represents common downregulated miRNAs between 6-h AMLO and 24-h Telmisartan (TELMI) treatments; the orange line represents common downregulated miRNAs among 6-h AMLO, MS, and TELMI treatments; the red line represents common downregulated miRNAs among 6-h AMLO, 6- and 24-h ENALA, and TELMI treatments.

The overall observations show that most of the differentially regulated miRNAs were unique with a few overlaps between drug and duration of treatment (Figure 2). hsa-miR-4497: MIMAT0019032 was commonly upregulated following 6-h AMLO and ENALA treatment, and the same treatments commonly downregulated hsa-mir-101-2:MI0000739. hsa-miR-365a-3p: MIMAT0000710 was upregulated upon 6-h AMLO treatment and downregulated upon 6-h MS treatment. AMLO treatment for 6 h and TELMI treatment for 24 h commonly downregulated hsa-miR-1277-5p: MIMAT0022724. AMLO treatment for 24 h and MS treatment for 6 h downregulated hsa-mir-1185-2:MI0003821. AMLO and ENALA 24-h treatment downregulated hsa-mir-12136:MI0039740. While hsa-miR-590-3p: MIMAT0004801 was downregulated by 6-h AMLO treatment, 6-h MS treatment, and 24-h TELMI treatment, the 6-h AMLO, 6-h and 24-h ENALA, and 24-h TELMI treatments downregulated hsa-mir-1277:MI0006419.

### 3.2. Differentially Expressed microRNAs and Their Target Genes

It is known that miRNA expression and their target gene expression have an inverse relationship. Therefore, it is critical to identify the target genes of these differentially expressed miRNAs to identify its probable impact on hypertension and side effects. The validated target genes from the miRwalk database were selected based on criteria, including binding to the 3′ untranslated regions (UTR) sites and a binding score ≥ 0.95 (Table S2). The downstream analysis of the target genes of the differentially regulated miRNAs on their implications with hypertension and related conditions was evaluated.

Among the validated target genes, the genes with the potential to be implicated with hypertension or related conditions were SH2B1, ADM2, VEGFA, ITGA1, IRS1, COL8A1, SMAD9, SMAD2, EDN1, CYP11B2, YES1, KCNK3, WNT2B, CHP1, PLCD3, PCDH10, FOXC1, SLC39A8, SOX6, TMEM33, FBXW7, TLR3, MAP2K4, ALDH1A3, RNASEH2B, ARMT1, AK4, and CDH2 (Table S3). A few genes targeted by the differentially regulated miRNAs were also associated with side effects as annotated by Human Phenotype Ontology (HPO) terms (Table S4). To validate these observations, a proteome-wide analysis upon treatment with various antihypertensives used in the protocol was performed.

### 3.3. Proteomic Changes Following Treatment

**Figure 3:**
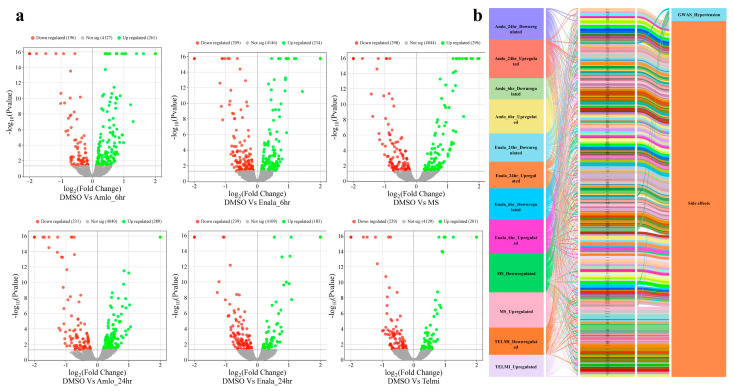
(**a**) Volcano plot representing significantly upregulated and downregulated proteins after each treatment (Amlodipine−AMLO; Enalapril−ENALA; Metoprolol−MS; Telmisartan−TELMI). (**b**) Alluvial plot representing proteins associated with hypertension and side effects.

A substantial number of proteins were differentially regulated after each treatment (Table S5), of which several were associated with hypertension and side effects in terms of HPO terms (Figure 3). AMLO treatment for 6-h significantly differentially regulated proteins, including 261 and 196 proteins, while for 24-h treatment, 289 and 231 were upregulated and downregulated, respectively. For 6-h ENALA treatment, differentially regulated proteins included 234 and 209 proteins, and for 24-h ENALA treatment, 183 and 239 were significantly upregulated and downregulated, respectively. MS treatment for 6- h differentially regulated proteins, including 296 and 298 proteins that were significantly upregulated and downregulated, respectively. TELMI treatment for 24-h differentially regulated proteins, including 201 and 229 proteins that were significantly upregulated and downregulated, respectively.

### 3.4. Coregulation of MiRNA Expression of Its Target Genes and Corresponding Proteomic Changes Following Treatment and Its Relationship to Hypertension HPO Terms

**Figure 4:**
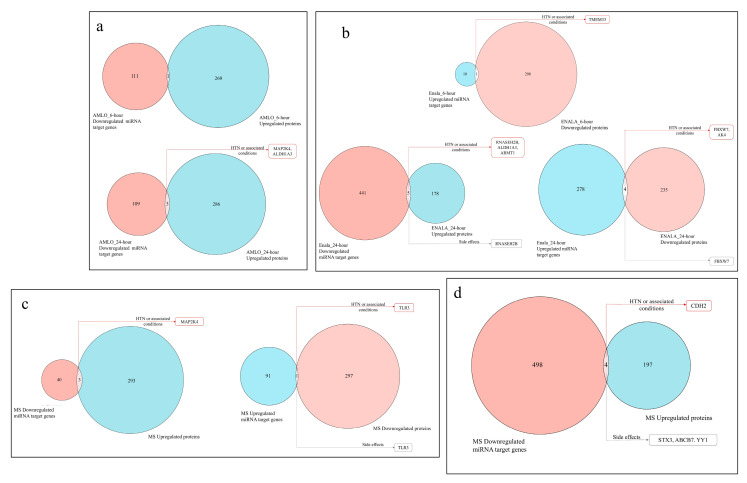
Venn diagrams showing the overlap between miRNA target genes of differentially regulated miRNA and proteins expressed following treatment. The proteins among the overlapping ones with a potential implication with hypertension or related conditions (marked in red box) and side effects (marked in grey box). (**a**) Amlodipine (AMLO) treatment for 6 h and 24 h, (**b**) Enalapril (ENALA) treatment for 6 h and 24 h, (**c**) Metoprolol (MS) treatment, (**d**) Telmisartan (TELMI) treatment.

It is known that miRNA expression and their target gene or protein expression have an inverse relationship. Therefore, it is critical to identify how these differentially expressed miRNAs impact protein expression and identify its probable impact on hypertension and side effects. The coregulation of miRNA expression of its target genes and proteins indicate that a large majority of the miRNA target genes do not overlap with the reciprocal target protein expression. Only a handful of proteins and miRNAs targets that are common reflect an inverse relationship, and interestingly, some of these do reflect miRNA-regulated proteins and are implicated either with hypertension or related conditions and with drug side effects (Figure 4).

**Figure 5:**
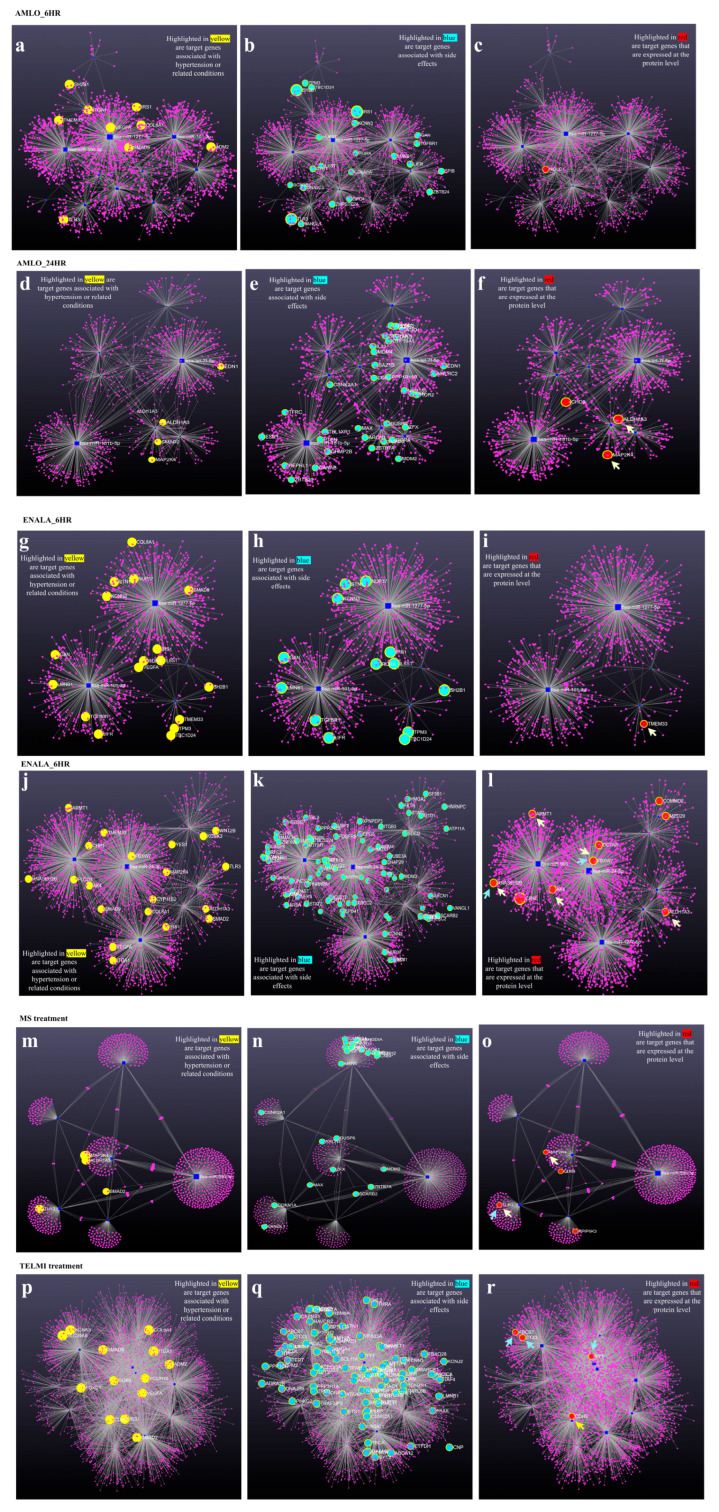
Network diagram showing drug-induced differentially expressed miRNAs and their respective target genes. Panels (**a**–**c**) show results for 6-h Amlodipine (AMLO) treatment; (**d**–**f**) for 24-h AMLO treatment; (**g**–**i**) for 6-h Enalapril (ENALA) treatment; (**j**–**l**) for 24-h ENALA treatment; (**m**–**o**) for Metoprolol (MS) treatment; and (**p**–**r**) for Telmisartan (TELMI) treatment. Dark blue nodes represent differentially regulated miRNAs; purple nodes represent target genes; yellow nodes denote target genes implicated in hypertension; cyan nodes indicate target genes with possible involvement in drug side effects; and red nodes represent target genes also reflected at the protein expression level. Yellow arrows highlight proteins implicated in hypertension, while blue arrows indicate proteins related to drug side effects.

The proteins that were upregulated due to miRNA downregulation were NOLC1 upon 6-h AMLO treatment, MAP2K4, ALDH1A3, and CHD9 upon 24-h AMLO treatment, ALDH1A3, RNASEH2B, DCTN5, ARMT1, and CRKL upon 24-h ENALA treatment, PPPIP5K2, MAP2K4, and CD99 upon 6-h MS treatment, and STX3, ABCB7, YY1, and CDH2 upon 24-h TELMI treatment. While evaluating the role of these genes in hypertension and HPO terms, we did find that the upregulation of proteins ALDH1A3, ARMT1, and CDH2 can be crucial in the treatment. In the above set of proteins, the MAP2K4 (Mitogen-Activated Protein Kinase Kinase 4)-coding gene for hypertension and RNASEH2B (ribonuclease H2 subunit) are the GWAS (genome-wide association study) genes for systolic and diastolic blood pressure. The proteins that were downregulated due to miRNA upregulation were TMEM33 upon 6-h ENALA treatment, TMEM33, FBXW7, COMMD2, MED29, and AK4 upon 24-h ENALA treatment, and TLR3 upon 6-h MS treatment. Among the downregulated proteins, TMEM33 and TLR3 may influence the therapy. FBXW7 and AK4 are GWAS genes for systolic and diastolic pressure. This is further demonstrated by the network interaction of validated miRNA target genes, and its potential impact on protein and their involvement in hypertension and drug induced side effects are denoted by their HPO terms (Figure 5).

The treatments and the protein implicated with side effects that are upregulated due to miRNA downregulation were RNASEH2B following 24-h ENALA treatment and STX3, ABCB7, and YY1 following TELMI treatment, and those that were downregulated due to miRNA upregulation were FBXW7 and TLR3 due to 24-h ENALA and MS treatment, respectively. None of the proteins upregulated following 6-h ENALA treatment reflected the reverse relation with ENALA-induced miRNA, and none of the proteins downregulated following AMLO or TELMI treatment were the result of miRNA upregulation.

## 4. Discussion

The study takes up an integrated omics-based analysis by considering the miRNA–proteome network approach to examine the influence of antihypertensive medications on miRNA expression and the proteome, which can eventually reflect the drug response or adverse effects. It is well established that microRNA-mediated regulation can play a crucial role at the post-transcriptional level. Studies have shown the role of miRNAs in hypertension development [12]. Thus, it was crucial to investigate the influence of antihypertensive drugs in modulating miRNAs associated with hypertension, and whether the miRNAs mediate drug side effects via HPO-linked pathways. The concentrations and duration used in the present study were selected based on the MTT assay and a preliminary study that included expression analysis of epigenetic regulators following drug treatment [14]. The results of small RNA sequencing provide proof that several miRNAs are differentially regulated upon antihypertensive treatment, providing insight to the possible role of miRNAs in antihypertensive drug response. Validated targets of the differentially regulated miRNAs were derived from the miRwalk database, and their implication in hypertension or related conditions were assessed, as differential expression of some of the target genes can result in adverse reactions and others result in a therapeutic response. The validation of target gene expression was followed up with proteome analysis. The expected inverse relationship between levels of drug-induced miRNA and their target proteins that are related to hypertension or associated conditions were observed. The protein Aldehyde Dehydrogenase 1 Family Member A3 (ALDH1A3) was increased upon AMLO and ENALA treatment for 24 h. A study showed the effect of cell type-specific ALDH1A3; upon upregulation, elevation of ALDH1A3 in endothelial cells supports their survival, while upregulation of ALDH1A3 in pulmonary arterial smooth muscle cells (PASMC) is reported to induce pathological proliferation and influence glycolytic metabolism, resulting in pulmonary arterial hypertension [20]. Our study showed that 24 h of AMLO and ENALA treatment increased the ALDHA13 level in endothelial cells, which indicates its impact on cell survival, but it can possibly have adverse effects on pulmonary arterial hypertension if the same result is replicated in PASMCs. Thus, the observation highlights the importance of evaluating the cell type-specific impact of the drugs and its clinical outcomes.

A high intracellular calcium level is associated with hypertensive individuals [21], and the elevated level of intracellular calcium is found in different cell types of hypertensive patients [22,23]. Transmembrane protein 33 (TMEM33) is known to modulate intracellular calcium homeostasis, thus acting as a regulator of the tubular endoplasmic reticulum network [24,25]. Studies have shown that TMEM33 plays a crucial role in vascular endothelial growth factor (VEGF)-mediated calcium signaling and, thus, in angiogenesis as well [26]. ENALA treatment for 6 h downregulates TMEM33, which can affect the intracellular calcium level, thus impacting treatment in hypertensive individuals. The present study also highlights that ENALA treatment can also be repurposed for cervical cancer treatment, as the increased level of TMEM33 was associated with increased tumor cell proliferation and poor prognosis in cervical cancer [27]. TMEM33 has also been reported to attenuate the cellular antiviral capacity of the host by blocking the IFN response. Here too, ENALA treatment can be repurposed for antiviral therapy or to increase the antiviral capacity by decreasing the level of TMEM33 through microRNA regulation [28].

Studies have shown that oxidative DNA damage is one of the crucial and initiating molecular mechanisms that leads to hypertension, and greater DNA damage was found in hypertensive patients with adverse effects, like coronary artery disease, concentric cardiac hypertrophy, and sustained or untreated hypertension [29]. The protein ARMT1 (Acidic Residue Methyltransferase 1), which was upregulated after 24-h ENALA treatment, is predicted to be involved in the DNA damage response (DDR). It exhibits cell-type dependent effects on survival with increased levels of the protein, promoting either enhanced or reduced survival depending on the cellular context [30]. Thus, if the increased level of ARMT1 due to ENALA treatment enhances DDR, the drug can have a protective effect on hypertension and related cardiovascular conditions; alternatively, the drug can also sensitize and cause an adverse effect.

CDH2, a N-Cadherin protein, was observed to be upregulated due to miRNA downregulation caused by TELMI treatment. The protein is involved in the assembly of vascular endothelial cadherin junctions, thereby limiting vascular permeability [31]. Restricting vascular permeability by increasing CHD2 can be protective against renal and cardiac damage in hypertensive patients, as vascular permeability is among the modifiable factors linked to the development of early manifestations of the above conditions [32]. An increase in vascular permeability is also an early symptom of endothelial dysfunction and atherosclerosis development [33]; TELMI treatment can thus be protective in these conditions as well.

The upregulation of a miRNA following MS treatment decreased the level of toll-like receptor-3 (TLR3). It has been reported that activation of TLR3 by double-stranded RNA can increase the inflammatory state and promote increased blood pressure and vascular dysfunction; hence, TLR3 can be a potential therapeutic target in treating hypertension [34]. Our study suggests that the decreased level of TLR3 upon MS treatment can potentially reduce TLR3-mediated inflammatory signaling, vascular dysfunction, and hypertension. Apart from the above 24-h AMLO treatment and MS treatment induced the upregulation of MAP2K4, a GWAS gene for hypertension. The upregulation of RNASEH2B, and downregulation of FBXW7 and AK4 all of which are GWAS genes for systolic and diastolic blood pressure following ENALA treatment for 24 h may have an influence in the therapeutic outcome. The identification of drug-induced miRNA changes in genes linked to hypertension provides mechanistic insights into how antihypertensive drugs exert their effects beyond their immediate pharmacological targets. The overlap with hypertension-implicated genes suggests that some of these epigenetic changes may contribute to the therapeutic efficacy or resistance observed in patients.

miRNA regulation also altered the levels of proteins that may be associated with the reported drug side effects in terms of HPO terms. ENALA treatment for 24 h upregulated RNASEH2B, which is associated with headache and glucose intolerance, and downregulated FBXW7 (F-box and WD repeat domain containing 7), which is associated with constipation, through miRNA regulation. miRNA regulation upon TELMI treatment upregulated the proteins STX3, YY1, and ABCB7. Upregulation of these proteins is related to potential side effects of antihypertensives. Along with vomiting, reported side effects of TELMI and STX3 are associated with pruritus and diarrhea. Similarly, along with the reported side effect tiredness, YY1 is related to sleep disturbance. ABCB7 is associated with depression, which is one of the side effects of TELMI. TLR3, which was downregulated by miRNA upregulation following MS treatment, is associated with tiredness, headache, and sleep disturbance, side effects caused by the drug. The association of differentially regulated genes and proteins with HPO terms for drug side effects offers a molecular basis for some of the adverse effects observed clinically. This information could be leveraged to predict or mitigate side effects in future drug development or personalized medicine approaches.

Thus, this study shows that antihypertensive drugs alter miRNA expression and their target proteins in human aortic endothelial cells, affecting therapeutic responses. The combined analysis of miRNAs and proteins reveals molecular changes linked to drug efficacy and side effects. This study has several strengths, including a comprehensive analysis of the modulation of miRNA and protein expression by antihypertensive drugs in an in vitro system. The integration of validated miRNA target predictions with proteome-wide validation enhances the biological relevance and robustness of the findings. By applying stringent target selection criteria and examining multiple drugs in a monotherapy regimen, this work provides valuable insights into the molecular mechanisms underlying therapeutic responses and potential adverse effects.

The limiting factor in the study is its use of in vitro models without clinical validation and selected doses and treatment times without comprehensive dose-response or time-course analyses. Additionally, healthy endothelial cells were used without hypertensive-related injury, which future work should address by incorporating disease-relevant models. However, it is difficult to perform similar study prospectively or retrospectively in clinical subjects, as the patients will be on multiple drugs. Considering this background, the present study may provide insight that can be validated in clinical subjects to identify drug-specific effects. Future studies can validate these key miRNA and protein biomarkers in patient samples or organotypic models to better reflect hypertension complexity. Further dose–response and time-course analyses, along with functional studies of critical miRNAs, will further clarify their roles and therapeutic potential

## 5. Conclusions

Our study integrates the miRNA and proteome data with GWAS genes or genes implicated with hypertension and HPO annotations, which provide a powerful framework for understanding both the beneficial and adverse effects of antihypertensive drugs at the molecular level. These insights may add to safer use and development of next-generation antihypertensive therapies with improved efficacy and safety profiles. These findings provide a foundation to prioritize miRNA and protein biomarkers for guiding and optimizing treatment strategies.

## Data Availability

All data pertaining to the work are included in the manuscript and supplementary files. The data are available from Xenod doi: https://www.mdpi.com/article/10.3390/cells14171359/s1.

## Supplementary Materials

The following supporting information can be downloaded at: https://www.mdpi.com/article/10.3390/cells14171359/s1. Figure S1: Methodology flow chart. Figure S2: MTT assay showing cell viability for different drug concentrations and time points. Table S1: Significantly regulated miRNAs (*p*-values < 0.05) with the respective fold change. Table S2: The validated target genes (3′ UTR binding, binding score ≥ 0.95) derived from the miRwalk database. Table S3: Validated miRNA target genes associated with hypertension. Table S4: Validated miRNA target genes and the side effects associated in terms of the HPO database. Table S5: Significantly differentially regulated proteins.
