# Supplementary figures and images for: Pharmacoepigenomic Impact of Antihypertensive Drugs on miRNome and Proteome and Its Potential Influence on Health and Side Effects
Source: Cells. 2025 Aug 31;14(17):1359. doi: 10.3390/cells14171359 (PMC12428730; doi:10.3390/cells14171359)

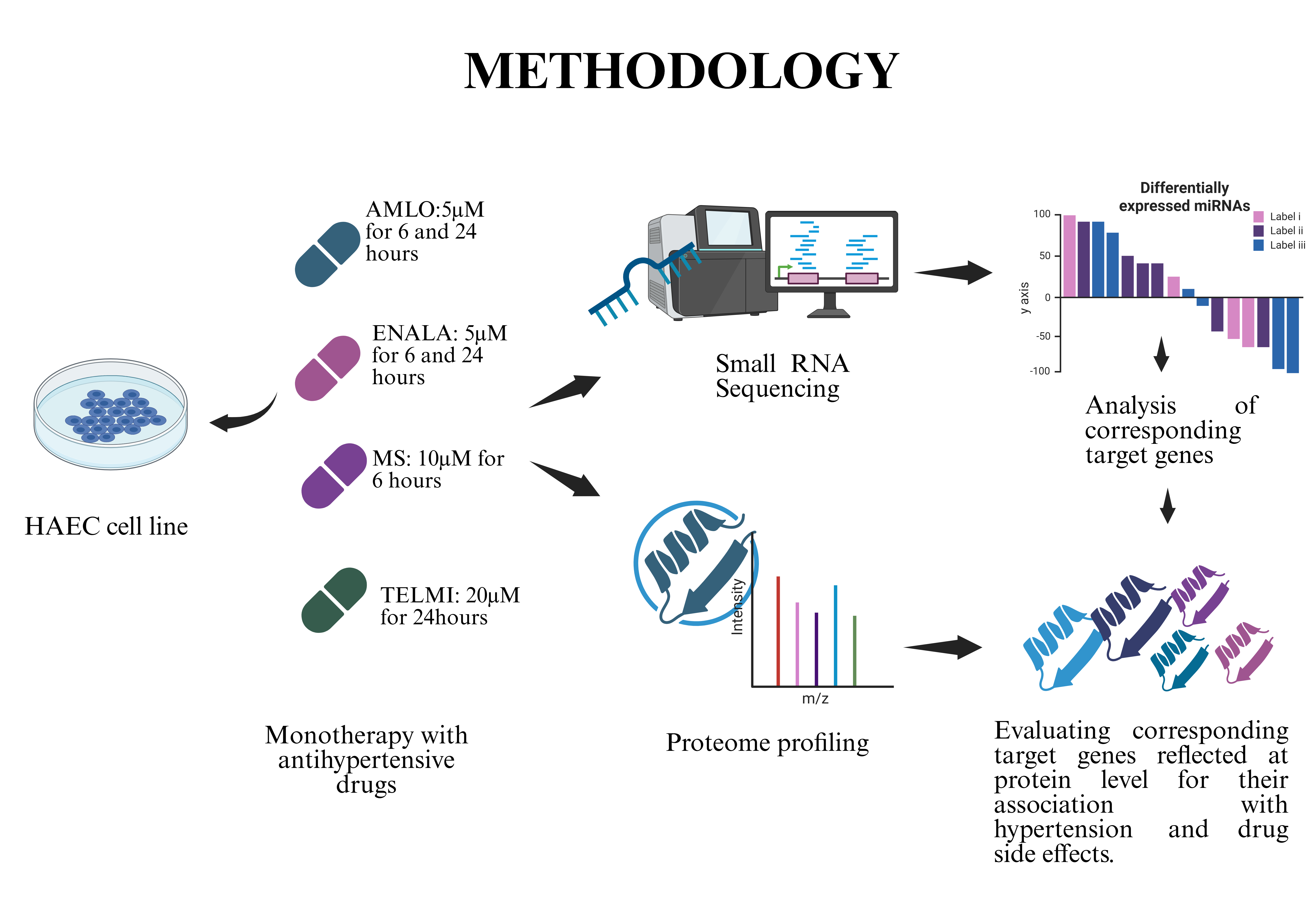

Supplement: Supplementary file 1 [file cells-14-01359-s001.zip › cells-3819120 supplementary/Figure S1 miR_methodology.jpeg]

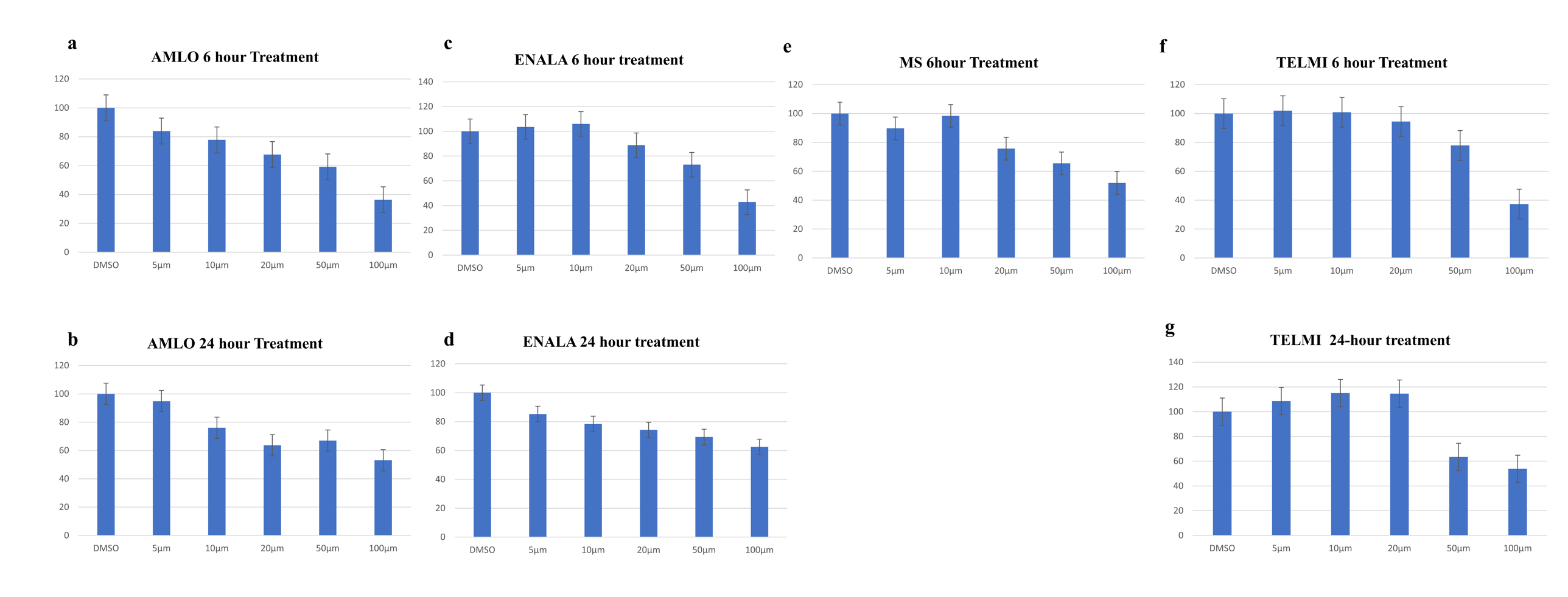

Supplement: Supplementary file 1 [file cells-14-01359-s001.zip › cells-3819120 supplementary/Figure S2 MTT graph.tif]
